# Antibacterial and white spot lesions preventive effect of an orthodontic resin modified with silver-nanoparticles

**DOI:** 10.4317/jced.58330

**Published:** 2021-07-01

**Authors:** Marco Sánchez-Tito, Lidia-Yileng Tay

**Affiliations:** 1 Facultad de Estomatología, Universidad Peruana Cayetano Heredia, Lima, Peru; 2 Facultad de Ciencias de la Salud, Universidad Privada de Tacna, Tacna, Peru

## Abstract

**Background:**

To evaluate the antibacterial property of a modified orthodontic resin with different concentrations of silver-nanoparticles (AgNPs), and quantify its preventive effect on the formation of white spot lesions (WSLs).

**Material and Methods:**

An orthodontic resin (Transbond XT) was modified with four concentrations of AgNPs (1%, 0.5%, 0.1%, and 0.05%), the orthodontic resin without AgNPs was used as control. Polymerized resin discs (n=80) were submitted to Agar diffusion test on Petri dishes inoculated with *Streptococcus mutans* and *Lactobacillus acidophilus*. In addition, resin discs of each group (n=40) were placed in 96-well plates with bacterial suspensions to evaluate the colony-forming-units (CFU). For the WSLs prevention test, brackets were bonded with the experimental orthodontic resins on 45 premolars (n=5), and were subjected to a microbiological caries induction method for 9 days. Photographs were taken before and after the test, and the images were evaluated with the Image J software to calculate the area of WSLs. The data were analyzed using ANOVA and Tukey-HSD test, Student´s t-test and Kruskal-Wallis test (α=0.05).

**Results:**

The 0.5% and 1% AgNPs modified resin inhibit the growth of *S. mutans* and *L. acidophilus*. All the modified resins showed significantly less CFU, when compared to the control (*p*<0.05). 1% AgNPs resin promote the higher prevention of WSLs formation. There was no significant difference between the control group and the 0.1% and 0.05% groups.

**Conclusions:**

0.5% and 1% of AgNPs modified orthodontic resin exhibit an important antibacterial activity against *S. mutans* and *L. acidophilus*, and prevent the formation of WSLs.

** Key words:**White spot lesions, antibacterial, orthodontics, adhesive, Silver-nanoparticles.

## Introduction

The most common complication in orthodontic treatment with fixed appliances is the high prevalence of demineralization of enamel caused by the bacterial biofilm accumulation around orthodontic brackets, particularly in the gingival margin, that can affect nearly 70% of the orthodontic patients (1,2). Poor oral hygiene can increase the colonization of *Streptococcus mutans* and *Lactobacillus acidophilus*, reducing the pH value to a critical level lower than 5.5, and promote the demineralization process leading the development of white spot lesions (WSLs) (3). In poor oral hygiene conditions WSLs can appear soon as one month after brackets bonding, and can compromise the patients´ aesthetics (3,4).

An appropriate oral hygiene is the most important preventive procedure to reduce the risk of WSLs formation, but it is known that there is a deficiency in many patients to follow the professional instructions to control biofilm formation (5). Other preventive strategies include the use of topical application of fluoride varnish, and the incorporation of antibacterial agents in mouthwashes and toothpastes; but these strategies depend on the patients´ collaboration (6,7). Thus, it has been proposed that the addition of antibacterial compounds into orthodontics bonding materials could overcome this problem, and require a minimum patient compliance (8).

These antibacterial compounds include the use of benzalkonium chloride, quaternary ammonium salts, and synthesized nanoparticles from silver, zinc oxide, cooper oxide, titanium dioxide, and others (9-13). On the other hand, the addition of antibacterial agents to orthodontics resins seems to decrease the bond strength to enamel, this effect is correlated to the concentration of the antibacterial agent (14).

The use of metallic nanoparticles has been proposed as an important resource to combat the formation of bacterial biofilm. Its antibacterial effectiveness has been related to the interaction with the bacteria membrane because of its size and high surface-volume ratio and not only due to metal ions release. Furthermore, bacteria have been shown to be less susceptible to develop resistance to metallic nanoparticles than to other antibacterial agents (15,16).

Silver-nanoparticles (AgNPs) have been used due to their antibacterial properties and low toxicity to human cells. Previous studies have shown that incorporating AgNPs to orthodontic resins has an important antibacterial effect, inhibiting the growth of *S. mutans* in *in vitro* models (11,12,17). However, to our knowledge its preventive effect on the formation of WSLs has not yet been reported.

The purpose of this study was to evaluate the antibacterial property of a modified orthodontic resin with different concentrations of AgNPs on the inhibition growth of *Streptococcus mutans* and *Lactobacillus acidophilus*, and quantify its preventive effect on the formation of WSLs.

## Material and Methods

-Ethical considerations and sample size 

This study was approved by the Institutional Committee of Research Ethics. Sample size was calculated using the Epidat® software Version 4.2 comparing the means of previous studies (8,11), with a confidence level of 95% and statistical power of 80%.

-Preparation of the AgNPs orthodontic resin

Light-cured orthodontic resin (Transbond XT; 3M Unitek, Monrovia, California, USA) was mixed with silver-nanoparticles (average particle size: 20 nm, purity: 99.99%; US Research Nanomaterials, Inc. Houston, TX, USA) at 1%, 0.5%, 0.1% and 0.05% concentrations (wt/wt). The preparation of the modified resin was made in a dark environment using a plastic spatula and a glass slab (11). To obtain 1% AgNPs modified resin, 0.03 g was mixed with 3 g of resin for 2 min to ensure a homogeneous distribution of the AgNPs. For 0.5%, 0.015 g of AgNPs were used; for 0.1% and 0.05%, 0.003 g and 0.0015 g of AgNPs were mixed with 3 g of orthodontic resin, respectively. The modified resins were stored in sterilized black syringes to prevent light transmission.

-Disc preparation

A total of 96 discs (5 mm diameter and 2 mm thickness) were prepared with the modified resins (24 discs for group), and additionally 24 discs were prepared with the conventional resin (control group). The discs were made filling a plastic mold with the resin, and it was covered with a celluloid strip and light-cured for 20 seconds from each side (Elipar DeepCure-L, 3M, St. Paul, MN, USA) (8,10). Then, the discs were removed, and the excesses were polished (Sof-LexTM, 3M ESPE, St. Paul, USA). Finally, the discs were sterilized with ultraviolet C (UVC) irradiation.

-Bacterial suspensions

*Streptococcus mutans* ATCC® 25175™ and *Lactobacillus acidophilus* ATCC® 4356™ (American Type Culture Collection, Manassas, VA, USA) were used. *S. mutans* was cultured in Brain Heart Infusion broth (BHI) and incubated at 37°C in microaerophilic condition (GaspakTM EZ Campy Container System) for 7 h to reach logarithmic phase. Then, an aliquot of the medium was transferred to a test tube containing physiologic serum, and the turbidity was adjusted to 0.5 McFarland standard (8). For *L. acidophilus*, De Man, Rogosa and Sharpe broth (MRS) was used, and the medium was prepared at the same conditions than *S. mutans*. The mediums were purchased from Liofilchem®.

-Agar diffusion test

For the bacterial inhibition growth test, 50 µL of *S. mutans* suspension was poured onto eight Petri dishes containing Brain Heart Agar (BHA), and one disc of each group were placed with 2-cm distance from each other (a total of 40 discs). The plates were incubated at 37°C in microaerophilic condition for 48 h. Then, the inhibition halos were measured using a digital caliper (Ubermann©) (8). The procedure was repeated for *L. acidophilus* using MRS Agar as growth medium.

-Colony forming units 

**Figure 1:**
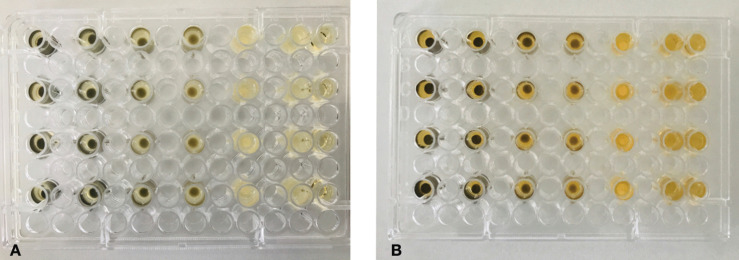
96-well plates with *S. mutans* (A) and *L. acidophilus* (B) suspensions for testing the antibacterial activity.

200 µL of BHI enriched with 1% sucrose and 15 µL of a suspension of *S. mutans* adjusted to 0.5 McFarland standard were poured in each well (columns: 1, 3, 5, 7, 11; and rows: A, C, E, G) of a sterile 96-well plate. In column 12, 200 µL of BHI was poured without the strain suspension (negative control). Discs of each group (a total of 20 discs) were placed in the wells and the plate was incubated at 37°C in microaerophilic condition for 24 h. Then, a dilution of 10-6 was made with 100 µL of the suspensions of each well with 900 µL of saline solution. 50 µL of the final dilutions were platted in BHA and incubated at 37°C for 48 h. The number of colony-forming-units (CFU/mL) were counted and transformed to log CFU per milliliter (18). The same procedure was repeated for *L. acidophilus* (Fig. 1).

-Bracket bonding procedure

Twenty-five human premolars, without cracks, fractures or caries lesions were used. Teeth were extracted for orthodontic reasons and collected from local clinics. Previous to bracket bonding, the enamel surface was polished with pumice and washed with distilled water. To standardize the exposed to etching and bonding, adhesive tape was place delimiting the area corresponding to the orthodontic bracket base, the bracket was positioned 2 mm gingivally to the buccal cusp tip and in the center of the clinical crown. The enamel was conditioned with 35% phosphoric acid (Unitek Etching Gel, 3M, Monrovia, USA) for 30 seconds and was washed for the double of time and dried with compressed air (12). Transbond XT Primer was applied to the enamel surface and light-cured for 20 seconds with an Elipar DeepCure-L unit. Five samples were used for bonding the brackets (Gemini, 3M Unitek, Monrovia, USA) with the each modified resin, additionally five samples were used to test the conventional resin. A constant force of 250 g was applied for 20 seconds to standardize the resin thickness, and the excess was removed with a scale. Finally, the samples were light-cured for 20 seconds (10), and stored in distilled water at 37°C for 24 h. Promptly, crowns and roots were sealed with an acid resistant nail varnish (Maybelline New York, NY, US) leaving a rectangular area of 2.5 mm x 2 mm in the cervical region of the bracket.

-Microbiological caries induction

A cariogenic solution was prepared as follow: 14.8 g BHI, 2 g yeast extract, 4 g glucose, and 8 g sucrose were mixed with 400 mL of distilled water and autoclaved at 121 °C for 15 min. Each tooth was placed in a test tube with 25 mL of the cariogenic solution inoculated with 25 µL of *S. mutans* and *L. acidophilus* suspensions. The tubes were incubated at 37°C for 9 days, the medium was changed every 48 h, without extra inoculation (19,20). After this period the nail varnish was removed with a scalpel blade and teeth were washed thoroughly with distilled water.

-Image acquisition 

Teeth were photographed in two times, T1: immediately after bracket bonding, and T2: after caries induction. Photograph acquisition was standardized following the recommendation of a previous study (21). Briefly, teeth were placed in a plastic test tube filled with silicon material (Zetaplus, Zhermack SpA, Italy) to ensure the correct position perpendicular to the camera lens. A special setting was constructed to standardize the photographic procedure attending the distance and angle of image acquisition, a rule was also placed in the setting to allow measurements. All photographs were taken with a Canon 60D camera (Canon Inc., Tokyo, Japan) with a 100 mm Macro Lens LF and a ring flash (Yongnuo Macro Ring Lite YN-14EX). The camera was set with an aperture of f9, a shutter speed of 1/125 s and an ISO sensitivity 200. Images were saved as JPEG files.

-Image analysis

All images were imported into Image J software (Image J, 1.52v for Mac OS, US National Institutes of Health, Bethesda, Md) and converted to 8-bit gray-scale. The scale was adjusted to pixel/mm for size measurement relative to the ruler of the photograph. The areas in T1 and T2 were selected with the freehand tool and the software was set to calculate the area expressed in mm2 (21).

-Statistical analysis

Data were analyzed by the SPSS software version 23.0 for Mac OS (SPSS Inc., IBM Corporation, NY, US). ANOVA was used to compare the antibacterial effect and the demineralization areas on enamel and Tukey-HSD test was used for pair comparison. Student´s t-test was used to compare the initial area and the demineralization zone. The Kruskal-Wallis test was used to analyze the UFC. A significance level of *p*<0.05 was adopted for all tests.

## Results

-Antibacterial effect

**Figure 2:**
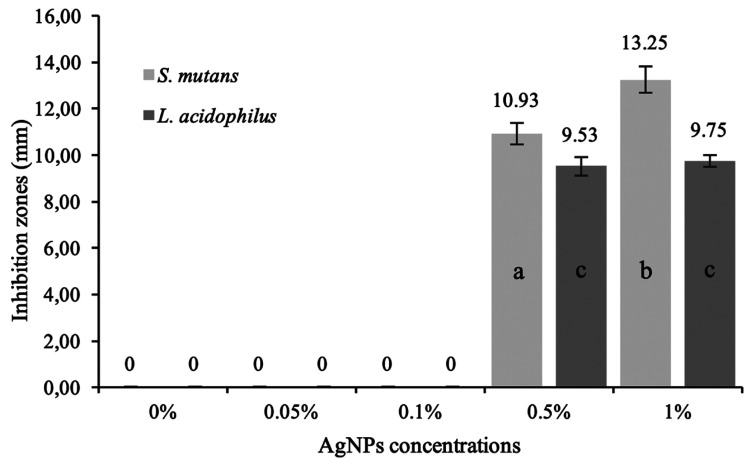
Inhibition zones by AgNPs concentrations. ANOVA test followed by Tukey-HSD test. Different letters indicate significant differences.

**Figure 3:**
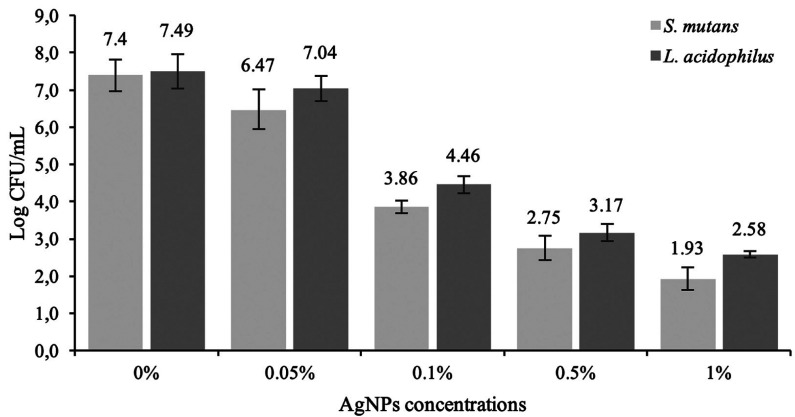
Colony-formation-units (Log CFU/mL) of Streptococcus mutans and Lactobacillus acidophilus.

Figure 2 shows the results of the agar diffusion test. Control group (0%) and the aggregate of 0.05% and 0.1 % of AgNPs showed no bacterial inhibition growth. The results showed a significant difference between 0.5% and 1% for *S. mutans* inhibition zones (*p*<0.05). There were no significant differences between the concentrations of AgNPs for *L. acidophilus* (*p*>0.05). On the other hand, all the modified resins showed significantly less CFU than the conventional resin for *S. mutans* and L. acidophilus (*p*<0.05) (Fig. 3).

-Enamel demineralization

**Figure 4:**
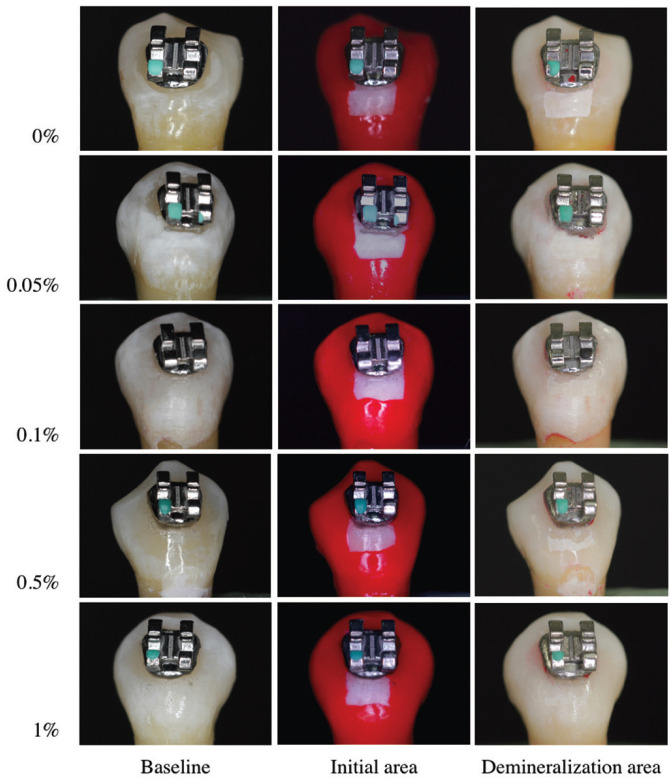
Samples of the different groups exposed to microbiological caries induction.

Student’s t test was used to compare the initial area and the enamel demineralization zone, all groups with AgNPs presented a lower demineralization area (*p*<0.05), the results are shown in Table 1 and Figure 4. In order to compare the enamel demineralization prevention effect of the modified resins with AgNPs, the differences between the initial and final areas were used. The one-way ANOVA test revealed significant difference between all groups (*p*<0.05); 1% AgNPs resin promote the higher prevention of WSL formation (3.21±0.56 mm2). Tukey-HSD test was used for the intragroup comparisons, the results showed significant difference between 1% AgNPs and the other groups. The 0.5% group was similar to the 0.1% group (*p*>0.05). There was no significant difference between the control group and the 0.1% and 0.05% groups (Table 2).

**Table 1:**
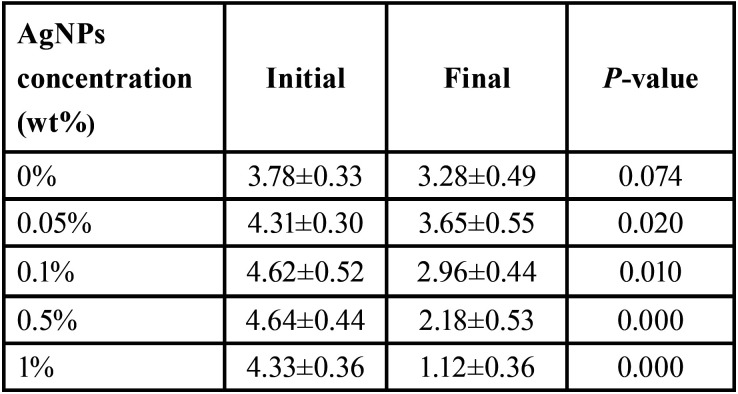
Comparison of the initial area and the enamel demineralization zone for the groups (mm2).

**Table 2:**
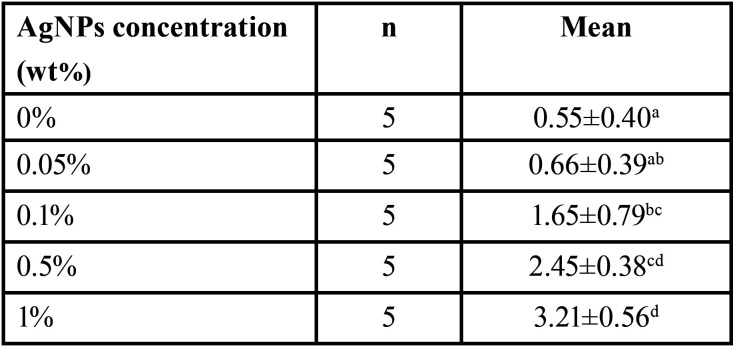
Enamel demineralization prevention areas of AgNPs modified resins. Means are expressed in mm2.

## Discussion

Demineralization of enamel around the brackets is a persistent problem in the orthodontic treatment, commonly associated to poor oral hygiene (3,4). A lot of strategies have been proposed to prevent its formation. Recently, the use of antibacterial compounds into the orthodontic resins had demonstrated to be effective to combat this problem, including the incorporation of different nanoparticles (9-13).

The first objective of this study was to evaluate the antibacterial effect of the aggregation of different concentrations of AgNPs into an orthodontic resin. The results demonstrated that the incorporation of 0.5 and 1% of AgNPs inhibit the growth of *S. mutans* and *L. acidophilus*, meanwhile lower concentrations such 0.05, 0.1% and the conventional resin had no antibacterial effect against the evaluated strains. In this study the highest concentration of silver-nanoparticles used was 1%, according to previous reports to avoid the reduction of mechanical and aesthetic properties of the resin when higher nanoparticles concentrations were used (11). The results showed a significant difference between 0.5% and 1% AgNPs for *S. mutans* inhibition zones; however, there were no differences between the same concentrations for *L. acidophilus*. On the other hand, all the resins with AgNPs concentrations showed significantly less colony forming units (CFU) than the control for *S. mutans* and *L. acidophilus* (*p*<0.05)

It has been proposed that the antibacterial mechanism of AgNPs is related to the positive charge on the Ag+ ion that allows the electrostatic attraction between the negative charge on the bacteria affecting the membrane-bound respiratory enzymes, destabilization of ribosomes, enzyme interaction promoting its damage and consequent cell death (22,24). Also, AgNPs possess the ability to alter the bacteria DNA inducing the loss of replication (24).

It has been demonstrated that when nanoparticles are smaller, their antibacterial effect is greater because of a higher surface/volume ratio (25), and this may be the explanation for the different results reported in some studies. Recently, Eslamian *et al*. (17) demonstrated that the incorporation of AgNPs with a particle size of 50 nm at a concentration of 0.3% into an orthodontic resin had an important activity against *S. mutans*, this antibacterial property was sTable for 30 days of evaluation. Yassaei *et al*. (11) carried out a study to evaluate the antibacterial effect of a modified orthodontic resin with copper oxide and silver oxide nanoparticles and reported that 1% concentration of these nanoparticles significantly reduced the growth of *S. mutans*, but at day 30 the number of colonies were no different from control group; it is important to point out that in this study the size of the nanoparticles was not indicated. Sodagar *et al*. (8) demonstrated that an orthodontic resin containing 5 and 10 % of silver/hydroxyapatite nanoparticles with a mean diameter of 55-65 nm reduce the growth of *S. mutans*, L. acidophilus and S. sanguinis. In contrast, Degrazia *et al*. (12) found that the incorporation of concentrations of 0.11%, 0.18% and 0.33% of AgNPs (particle size <150 nm) into an orthodontic primer has no effect on the growth inhibition of *S. mutans* in direct contact assay, meanwhile the same concentrations demonstrated an important antibacterial activity in liquid medium, the authors explain that these results can be due to a homogeneous dispersion of the nanoparticles in the liquid medium that enhance its properties. In our study the size of the AgNPs was 20 nm, this may explain the significant antibacterial activity observed against *S. mutans* and *L. acidophilus*, both in the disk diffusion assay and the UFC.

The evaluation of WSLs has been carried out with different methods such as light-induced fluorescence (QLF), and laser fluorescence in clinical and *in vitro* studies (19,26); but these methods represent a high cost in equipment acquisition and relative complicate procedures. The use of digital photographs has been proposed by some authors, indicating a high degree of accuracy and reproducibility, and represent an inexpensive method for quantification of demineralized areas on dental surface (21,27). Thus, the second objective of this study was to evaluate the effect of the incorporation of AgNPs into an orthodontic resin on the prevention of WSLs formation. The results demonstrated that the incorporation of 0.1% or a higher concentration of AgNPs were effective to prevent the demineralization of the enamel and consequently avoided the WSL formation. Ahn *et al*. (28) demonstrated that the incorporation of silica nanofillers and different concentrations of AgNPs with a size less that 5 nm can prevent enamel demineralization around brackets without decreasing its mechanical properties. Since the size of the AgNPs used in this study remained small (20 nm), it is possible that the preventive effect on demineralization may have been effectively promoted.

There is no standard protocol for the incorporation of AgNPs into orthodontic resins, and some factors may be considered such the volume and size of the nanoparticles, or the addition to the resin or primer, and the potential of these nanoparticles to modified the mechanical and aesthetic properties of the orthodontic resins. Also, all studies have been carried out an *in vitro* models, being necessary to perform clinical trials to evaluate its clinical performance, and the risks and benefits of adding AgNPs into orthodontic resins to prevent the formation of WSLs.

## Conclusions

The incorporation of 0.5% and 1% of AgNPs into orthodontic resin provide and important antibacterial activity against *S. mutans* and *L. acidophilus*, and prevent the formation of white spot lesions.
